# Supplementary material for: High-fidelity single-shot readout of single electron spin in diamond with spin-to-charge conversion
Source: Nat Commun. 2021 Mar 9;12:1529. doi: 10.1038/s41467-021-21781-5 (PMC7943573; doi:10.1038/s41467-021-21781-5)
Supplement: Supplementary file 1 — Supplementary Information [file 41467_2021_21781_MOESM1_ESM.pdf]

Supplementary Information for High-fidelity single-shot readout of single electron spin in diamond with spin-to-charge conversion

Qi Zhang[1,2,3*], Yuhang Guo[1,2,3*], Wentao Ji[1,2,3*], Mengqi Wang[1,2,3], Jun Yin[1,2,3], Fei Kong[1,2,3], Yiheng Lin[1,2,3], Chunming Yin[1,2,3], Fazhan Shi[1,2,3], Ya Wang[1,2,3†], Jiangfeng Du [1,2,3†]
[1] Hefei National Laboratory for Physical Sciences at the Microscale and Department of Modern Physics, University of Science and Technology of China, Hefei 230026, China.
[2] CAS Key Laboratory of Microscale Magnetic Resonance, University of Science and Technology of China, Hefei 230026, China.
[3] Synergetic Innovation Center of Quantum Information and Quantum Physics, University of Science and Technology of China, Hefei 230026, China.
[*] These authors contributed equally to this work.
[†] E-mail: ywustc@ustc.edu.cn, djf@ustc.edu.cn

EXPERIMENTAL APPARATUS

All the experiments in this work are performed on a home-built low-temperature ODMR setup. The sample is hosted at the temperature of 8 K in a closed-cycle optical cryostat (Montana Instruments Cryostation S200). The sample is positioned by a XYZ piezo stack (Attocube), at the focal point of a 0.9 NA objective (Olympus MPLFLN100x). The objective is mounted on the side wall of the vacuum chamber. A feedback loop is used to stabilize the temperature of the objective at room temperature. We use three lasers to excite the NV center: a traditional 532 nm laser (Changchun New Industries Optoelectronics Technology) to reset charge state and two tunable 637 nm lasers (New focus TLB-6704-OI, Toptica DLC DL PRO HP 637) to perform E_y and $E_{1,2}$ resonant excitation respectively. The wavelengths of the 637 nm lasers are stabilized using a wavemeter (HighFiness WSU-10). We use a 1064 nm laser (Changchun New Industries Optoelectronics Technology MSL-III-1064) to ionize the NV center. A permanent magnet is positioned near the sample by another set of XYZ piezo stack (Attocube) to produce a static magnetic field. Microwave pulses are generated by an arbitrary waveform generator (Keysight M8190A). After amplification (minicircuit), the Microwave pulses are fed to a gold strip line fabricated on top of the sample.

SAMPLE INFORMATION

Here we use a chemical vapor deposition grown diamond with (111) surface. The NV center studied here is 9 μm below the diamond surface. The NV axis is perpendicular to the diamond surface. A solid immersion lens (SIL) is wrote with focused ion beam system to enhance the photon collection efficiency[1, 2]. Gold microwave strip line and electrodes are deposited around the SIL (Fig. S1ab). Fluorescence saturation curves under resonance excitation are shown in Fig. S1cd. The fluorescence count rate (I) is the peak count rates of the photoluminescence (PL) decay curves (Fig. 1d of the main text) under different excitation powers (P). The data are fit with $I = I_{max}/(1 + P/P_0)$, where P_0 is the saturation power and I_{max} the maximum count rate.

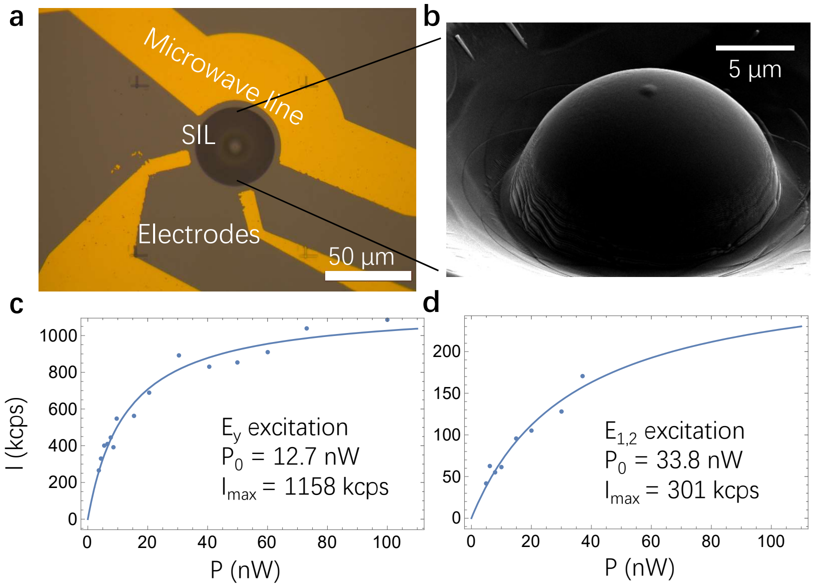

FIG. S1. a. Bright field microscopy photograph of the SIL. Electrodes keep grounded in this work. b. Electron microscopy image of the SIL. c. Fluorescence saturation curves under E_y excitation, with saturation power P_0 = 12.7 nW and maximum count rate I_{max} = 1158 kcps. d. Fluorescence saturation curves under $E_{1,2}$ excitation, with saturation power P_0 = 33.8 nW and maximum count rate I_{max} = 301 kcps.

SIL is known to enhance both the collection efficiency and laser pumping efficiency. In Fig. S2 we evaluate the pumping enhancement by comparing the saturation powers of NV centers with SIL and planar surface. The experiment is conducted with 532 nm excitation at room temperature. We measure the fluorescence saturation curves of NV centers on another diamond with (111) surface. The NV centers are all oriented along the [111] direction perpendicular to diamond surface. The NV depth is obtained by multiplying the diamond refractive index (2.4) by the moving distance of nanoscanner from the NV center to the diamond surface. The saturation power shows a negative correlation with the NV depth (Fig. S2b). For a 2.4μm-deep NV center the saturation power is 424 μW, 2.3 times of that with SIL. This enhancement should also hold for 1064 nm. According to the results in Fig. 2g of main text, the ionization rate is proportional to the NIR power density. This effect can be compensated by

directly increasing the NIR power for applications on planar diamond. Nano-pillar is an alternative to maintain high pumping efficiency for shallow NV centers.

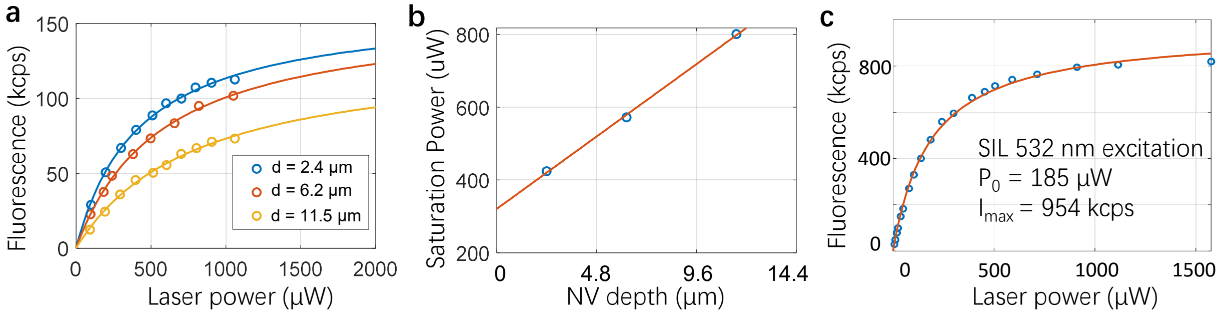

FIG. S2. 532 nm laser pumping efficiency enhancement of SIL over planar diamond. a. Fluorescence saturation curves for NV centers with different depth beneath a planar (111) diamond surface. b. Saturation power dependence on NV depth. c. Florescence saturation curve of the NV center with SIL under green laser.

SPIN STATE INITIALIZATION FIDELITY

To estimate the spin initialization fidelity, we prepare $|0\rangle$ ($|\pm1\rangle$) state by a $E_{1,2}$ (E_y) pulse and record the resonance fluorescence time trace under E_y ($E_{1,2}$) illumination. We fit the results with double exponential decay curves and extract the initial and final equilibrium count rate. The initial count rate for $|0\rangle$ ($|\pm1\rangle$) state is 166.7 kctps (39.4 kctps). Note that final count rate of the $|\pm1\rangle$ result is higher than the $|0\rangle$ result. This is due to a higher background count rate from the $E_{1,2}$ laser (2.8 kctps) than from the E_y laser (0.5 kctps). After subtracting the background, we can estimate a remaining population of 0.17$\pm$0.06 % in $|0\rangle$ state after E_y pumping, and 0.06$\pm$0.15 % in $|\pm1\rangle$ state after $E_{1,2}$ pumping, giving state initialization fidelity of 99.83$\pm$0.06 % for $|\pm1\rangle$ state and 99.94$\pm$0.15 % for $|0\rangle$ state. In the main text, to initialize to $|0\rangle$ state, we apply a 20 μs $E_{1,2}$ pulse, corresponding to a 99.82% initialization fidelity. To initialize to $|+1\rangle$ state, along with the E_y laser pumping $|0\rangle$ state, we apply an additional microwave pulse to flip the population from $|-1\rangle$ back to $|0\rangle$ state. After 200 μs E_y and microwave pulse, we can estimate the remaining population in both $|0\rangle$ and $|-1\rangle$ as 0.17%, giving 99.66% initialization fidelity into $|+1\rangle$ state.

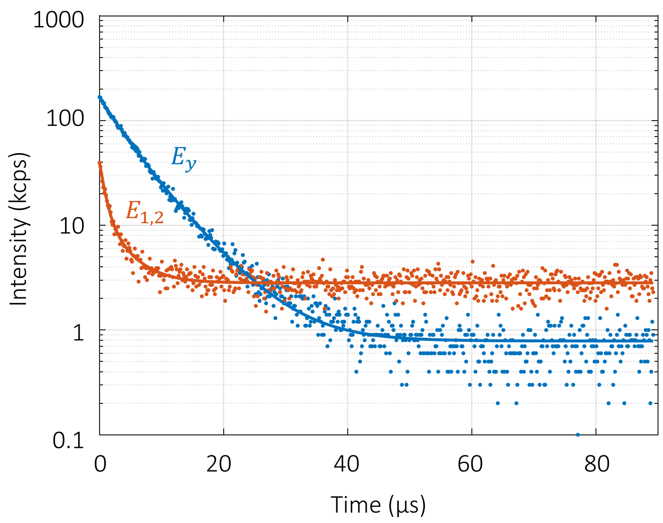

FIG. S3. Photoluminescence decay of NV center, initially prepared in $|0\rangle$ (blue) and $|\pm1\rangle$ (orange).

IMPROVING CHARGE READOUT FIDELITY

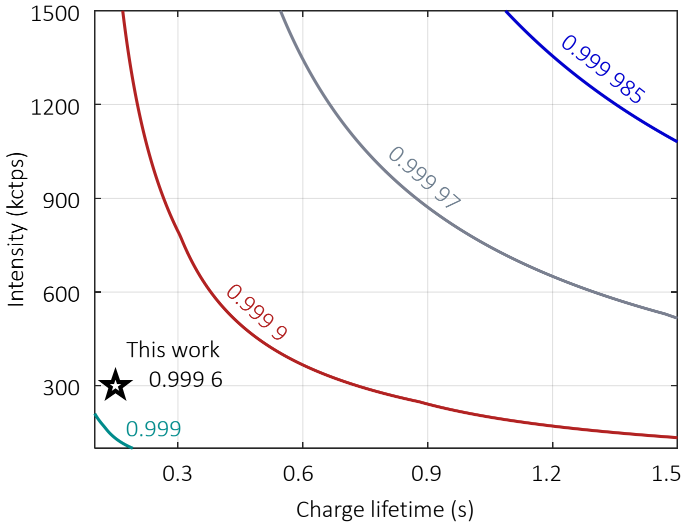

FIG. S4. Effect of photon count rate and charge lifetime on non-demolition charge readout fidelity.

The charge readout fidelity mainly depends on the photon count rate and charge lifetime. Resonance fluorescence count of NV^- is proportional to spontaneous emission rate and fluorescence collection efficiency, while NV^0 count is mainly affected by background fluorescence. Here we assume a background fluorescence of 2.5 kctps, and optimize the readout window by balancing the effects of photon shot noise and charge state lifetime, to obtain the best non-demolition charge readout fidelity. The black star marked our current level of charge readout (fidelity 99.96%). For single-shot charge readout, higher fidelity could be achieved by prolonging the reading time.

MODEL FOR SPIN AND CHARGE DYNAMICS

In order to understand the charge dynamics observed in experiment, we consider a 7 level energy diagram relevant to the SCC process, as depicted in Fig.S3. The dynamics among different levels can be described by the following rate equation

$$\begin{aligned}
\frac{d}{dt}P_0 &= -\Gamma_{ex,0}P_0 + \Gamma P_{E_y} + \Gamma_{isc,0}P_{singlet} + \Gamma_{flip,E_{1,2}}P_{E_{1,2}} \\
\frac{d}{dt}P_{+1} &= -\Gamma_{ex,\pm1}P_{+1} + 0.5\Gamma P_{E_{1,2}} + \alpha\Gamma_{flip,E_y}P_{E_y} + \Gamma_{isc,+1}P_{singlet} \\
\frac{d}{dt}P_{-1} &= -\Gamma_{ex,\pm1}P_{-1} + 0.5\Gamma P_{E_{1,2}} + (1-\alpha)\Gamma_{flip,E_y}P_{E_y} + \Gamma_{isc,-1}P_{singlet} \\
\frac{d}{dt}P_{E_y} &= \Gamma_{ex,0}P_0 - \Gamma P_{E_y} - \Gamma_{isc,E_y}P_{E_y} - \Gamma_{ion}P_{E_y} + \Gamma_{flip,E_y}P_{E_y} \\
\frac{d}{dt}P_{E_{1,2}} &= \Gamma_{ex,\pm1}(P_{+1}+P_{-1}) - \Gamma P_{E_{1,2}} - \Gamma_{isc,E_{1,2}}P_{E_{1,2}} - \Gamma_{flip,E_{1,2}}P_{E_{1,2}} \\
\frac{d}{dt}P_{singlet} &= \Gamma_{isc,E_y}P_{E_y} + \Gamma_{isc,E_{1,2}}P_{E_{1,2}} - (\Gamma_{isc,0} - \Gamma_{isc,+1} + \Gamma_{isc,-1})P_{singlet} \\
\frac{d}{dt}P_{NV^0} &= \Gamma_{ion}P_{E_y}
\end{aligned}$$

where the spontaneous emission rate $\Gamma = 77$ MHz [3], the excitation rate $\Gamma_{ex,0}$ is estimated directly according to the fluorescence saturation curve, $\Gamma_{ex,0} = \frac{\mathrm{PL}}{\mathrm{PL_{sat}-PL}}\Gamma = \frac{480}{1157-480} * 77 \sim 54$ MHz. $\Gamma_{isc,+1} + \Gamma_{isc,-1} + \Gamma_{isc,0} = 1/\tau_{singlet} \sim 0.3$ MHz is the singlet decay rate at 8K [4]. The branching ratio from the singlet state to the ground state is $\Gamma_{isc,+1} : \Gamma_{isc,-1} : \Gamma_{isc,0}$ = 1:1:8 according to the literature [5]. With these fixed parameters, we further determine the rates $\Gamma_{ex,0}$, Γ_{isc,E_y} and Γ_{flip,E_y} by fitting the fluorescence decay under E_y excitation. Noted that since $|\pm1\rangle$ is not excited under E_y illumination, the rates

TABLE I. Ionization rates in Fig. 2fg

Effective NIR laser power [mW]	Γ_{ion} [MHz]
7.0	0.52 ± 0.01
12.0	0.81 ± 0.01
18.4	1.23 ± 0.02
28.0	2.21 ± 0.05
39.8	2.67 ± 0.07
45.0	2.79 ± 0.08

TABLE II. Best-fit parameters

Parameter	Value	Data source
$\Gamma_{ex,0}$	54.906 ± 0.372 MHz	E_y saturation curve (Fig. S1a)
Γ_{flip,E_y}	0.752 ± 0.005 MHz	E_y PL decay curve (Fig. 1d)
Γ_{isc,E_y}	0.132 ± 0.004 MHz	
$\Gamma_{ex,\pm1}$	1.209 ± 0.449 MHz	$E_{1,2}$ saturation curve (Fig. S1b)
$\Gamma_{flip,E_{1,2}}$	2.010 ± 4.277 MHz	$E_{1,2}$ PL decay curve (Fig. 1d inset)
$\Gamma_{isc,E_{1,2}}$	52.760 ± 0.376 MHz	
α	0.275 ± 0.191	SCC curves (Fig. 3b)

$\Gamma_{ex,\pm1}$, $\Gamma_{isc,E_{1,2}}$ and $\Gamma_{flip,E_{1,2}}$ are set to zero in the PL decay simulation of E_y excitation. Similarly, the rates $\Gamma_{ex,\pm1}$, $\Gamma_{flip,E_{1,2}}$ and $\Gamma_{flip,E_{1,2}}$ are determined by fitting the fluorescence decay under $E_{1,2}$ excitation. With all these parameters derived, the ionization rates Γ_{ion} are then obtained by fitting the charge conversion curves in Fig. 2f. The derived ionization rates are given in Table I. The branching factor α of the spin-flip process from $|E_y\rangle$ to $|\pm 1\rangle$ (Fig. S5) is determined by fitting the pulsed SCC curve in Fig. 3b. The origin of this spin-flip asymmetry is currently unclear and may be related to strain. All the parameters derived with our model are given in Table II.

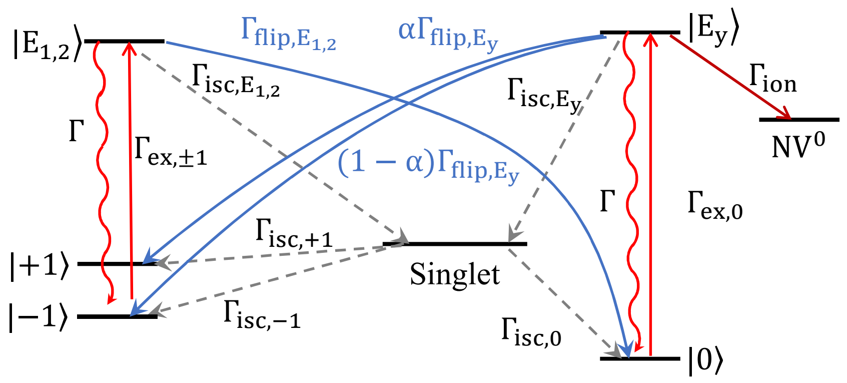

FIG. S5. Detailed energy level diagram used in the simulation. The optical transitions are denoted by red arrows. The ionization process is denoted by dark red arrow. The spin flip transitions are denoted by blue arrows. The inter-system crossing(ISC) transitions are denoted by dashed grey arrows.

FIDELITY OF THE RESONANCE FLUORESCENCE METHOD

The fidelity of the resonance fluorescence method can be modeled by considering the photon number statistics. Here we consider a readout threshold of one photon, which is adopted in the main text. The fidelity of the $m_s = 0$ state readout is the probability of collecting at least one photon ($n \geqslant 1$), conditional on the bright initial state, i.e. $F_0 = P(n \geqslant 1|m_s = 0) = 1 - P(n = 0|m_s = 0)$. Similarly, the fidelity of the $m_s = \pm1$ state readout is $F_1 = P(n = 0|m_s = \pm1)$.

If the count rate is constant, i.e. spin flip is ignored, in a time duration t, the collected photon number for both the $m_s = 0$ and $m_s = \pm1$ states yield a Poisson distribution. The probability of collecting zero photon during time t for $m_s = 0$ and $m_s = \pm1$ state are given by $Q_{0,1}(t) = e^{-a_{0,1}t}$, with a_0 and a_1 denoting the photon count rate of $m_s = 0$ and $m_s = \pm1$ state respectively. Note that a_0 corresponds to the initial count rate of the PL decay curve (Fig.1d of the main text). The spin flip

process is characterized by the PL decay rate γ, which shouldn't be confused with the spin-flip rate Γ_{flip}. The spin-flip rate Γ_{flip} characterizes the intrinsic spin-flip process, whereas the PL decay rate γ depends on both the spin-flip rate and the excitation strength. The dependence of decay rate on the spin-flip rate and the excitation strength can be inferred from the model given in the previous section. After an excitation of duration t, the probability of the spin remaining not flipped is given by $P_0(t) = e^{-\gamma t}$. In the whole readout time window t, for the $m_s = 0$ state, there is a finite probability of spin flipping, hence $P(n = 0|m_s = 0)$ consists of two parts: (i) the spin is not flipped during time t, and (ii) the spin is flipped at time $\tau \in [0, t]$. This leads to

$$\begin{aligned} P(n = 0|m_s = 0) &= Q_0(t)P_0(t) + \int_0^t \gamma \mathrm{d}t P_0(\tau)Q_0(\tau)Q_1(t-\tau) \\ &= \frac{a_0 - a_1}{a_0 - a_1 + \gamma} e^{-(a_0+\gamma)t} + \frac{\gamma}{a_0 - a_1 + \gamma} e^{-a_1 t}. \end{aligned}$$

For the dark state, the spin-flip can be ignored, giving

$$P(n = 0|m_s = \pm 1) = e^{-a_1 t}.$$

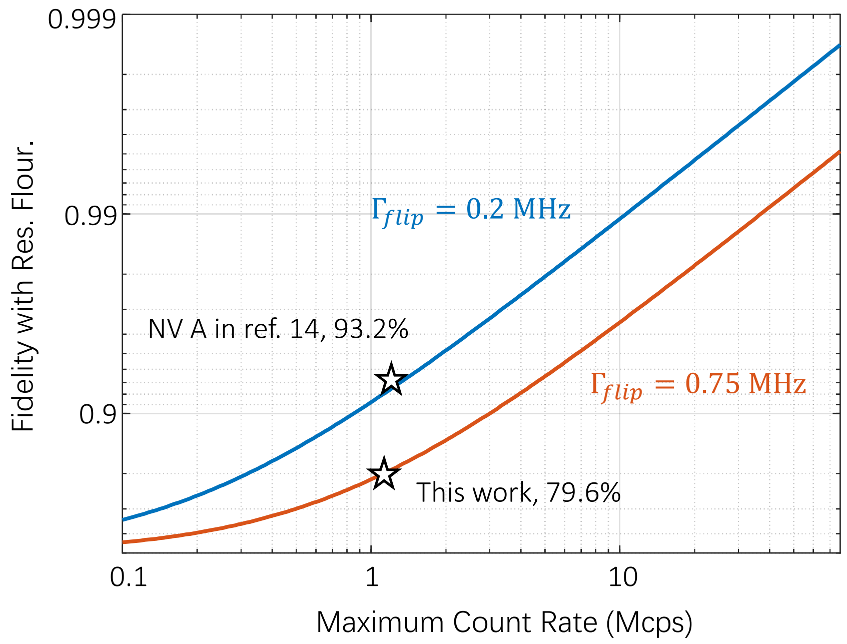

FIG. S6. Optimal fidelity of the resonance fluorescence method. The orange line corresponds to the spin-flip rate observed in this work. The blue line corresponds to a previously reported NV center with a lower spin-flip rate [6]. The observed saturation counts and readout fidelity for both works are marked with asterisks.

The average fidelity is given by the average of bright and dark state readout fidelity, i.e. $F_{avg} = (F_0 + F_1)/2$. F_{avg} is optimized at readout window

$$t_{opt} = \frac{\ln(a_0 + \gamma) - \ln(a_1)}{a_0 - a_1 + \gamma}.$$

The optimal fidelity is

$$F_{opt} = \frac{1}{2}\left[1 + \frac{a_0 - a_1}{a_0 - a_1 + \gamma}\left(\left(\frac{a_0 + \gamma}{a_1}\right)^{-\frac{a_1}{a_0 - a_1 + \gamma}} + \left(\frac{a_0 + \gamma}{a_1}\right)^{-\frac{a_0 + \gamma}{a_0 - a_1 + \gamma}}\right)\right]. \tag{1}$$

To compare optimal fidelity under different saturation counts and spin-flip rate, we take the NV centers in Fig. 4b of the main text as examples: (i) the NV center used in this work, with saturation counts of 1.1 Mcps and spin-flip rate of $\Gamma_{flip} = 0.75$ MHz, (ii) a previously reported NV center, with saturation counts of 1.2 Mcps and spin-flip rate of $\Gamma_{flip} = 0.2$ MHz [6]. We also assume

that, for both NV centers, the counts in $m_s = \pm 1$ state is $a_1 = 1$ Kcps. The optimal fidelity of the resonance fluorescence method is calculated using Eq. 1 and depicted in Fig. S6.

To have a better understanding of Eq. 1, we take the limit of F_{opt} as $a_1 \to 0$, since in our experiments, both a_0 and γ is in the order of MHz, while a_1 is in the order of kHz. This gives

$$\lim_{a_1 \to 0} F_{opt} = \frac{1}{2}\left(1 + \frac{a_0}{a_0 + \gamma}\right). \tag{2}$$

Note that the only independent parameter here is the ratio between a_0 and γ, hence we introduce $n_0 = a_0/\gamma$, which gives the average total photon number of the $m_s = 0$ state. Rewriting Eq. 2 gives

$$F_{opt} = 1 - \frac{1}{2(1 + n_0)}. \tag{3}$$

This clearly shows that, for the resonance fluorescence method, the optimal fidelity is limited by n_0, which is essentially determined by the saturation counts of the NV center and the intrinsic spin-flip rate Γ_{flip}.

[1] Hadden, J. P. *et al.* Appl. Phys. Lett. 97, 241901 (2010).
[2] Siyushev, P. *et al.* Appl. Phys. Lett. 97, 241902 (2010).
[3] I. Meirzada, Y. Hovav, S. A. Wolf, and N. Bar-Gill, Phys. Rev. B 98, 245411 (2018).
[4] L. Robledo, H. Bernien, T. van der Sar, and R. Hanson, New J. Phys. 13, 025013 (2011).
[5] N. Kalb, P.C. Humphreys, J.J. Slim, and R. Hanson, Phys. Rev. A 97, 062330 (2018).
[6] L. Robledo, L. Childress, H. Bernien, B. Hensen, P. F. A. Alkemade, and R. Hanson, Nature 477, 574578 (2011).
